# ANALYTICAL CONSIDERATIONS OF DEVELOPING A PHENOTYPIC AGING MEASURE: THE CONCEPTUAL FRAMEWORK MUST COME FIRST!

**DOI:** 10.1093/geroni/igz038.2148

**Published:** 2019-11-08

**Authors:** Michelle Shardell, Michelle Shardell, Pei-Lun Kuo, Jennifer Schrack, Eleanor M Simonsick, Susan M Resnick, Morgan E Levine, luigi Ferrucci

**Affiliations:** 1 Translational Gerontology Branch, Intramural Research Program, National Institute on Aging, National Institutes of Health, Baltimore, Maryland, United States; 2 National Institute on Aging, Baltimore, Maryland, United States; 3 Johns Hopkins University Bloomberg School of Public Health, Baltimore, Maryland, United States; 4 Yale School of Medicine, New Haven, Connecticut, United States

## Abstract

We propose a latent structural model framework where phenotypic aging is a latent variable influenced by chronological age, genes and environment. Within this framework, phenotypic age influences aging-related outcomes and is reflected by latent domains of aging (body composition, energetics, homeostasis, and neural functioning) reflected by biomarkers. First, we validate the framework by selecting age-associated domain-specific biomarkers and assessing internal consistency and convergent construct validity (Cronbach’s alpha). Using data from the Baltimore Longitudinal Study of Aging, within-domain Cronbach’s alphas ranged from 0.80 to 0.92, supporting convergent construct validity. Second, we evaluate two broad methods for combining biomarkers into one phenotypic age measure customized to different objectives: 1) confirmatory factor analysis of chronological age-adjusted biomarkers to create a measure to identify pleiotropic genetic and environmental mechanisms, and 2) machine-learning methods to create a measure optimizing predictive and concurrent criterion validity. This framework will enable evaluation of candidate biological mechanisms of aging.

